# Correction: Pollen morphology of Polish species from the genus *Rubus* L. (Rosaceae) and its systematic importance

**DOI:** 10.1371/journal.pone.0237625

**Published:** 2020-08-07

**Authors:** Kacper Lechowicz, Dorota Wrońska-Pilarek, Jan Bocianowski, Tomasz Maliński

Incorrect funding information is included in the Acknowledgments. The correct funding information should be included in the Funding statement and read as follows: The publication was co-financed within the framework of the Ministry of Science and Higher Education program as "Regional Initiative Excellence" in years 2019–2022 (Project No. 005 / RID / 2018/19). No additional external funding was received for this study. The funders had no role in study design, data collection and analysis, decision to publish, or preparation of the manuscript.
